# An Interpretable Center-Specific Machine Learning Model for Risk Stratification Following Mitral Valve Surgery: A Pilot Study

**DOI:** 10.3390/jcm15145496

**Published:** 2026-07-13

**Authors:** Aleksandra Stańska, Miriam Kilarska, Mateusz Janeczek, Wojciech Karolak, Andrzej Klapkowski

**Affiliations:** 1 Division of Quality of Life Research, Department of Psychology, Faculty of Health Sciences, Medical University of Gdańsk, 80-210 Gdańsk, Poland; 2 Department of Cardiac & Vascular Surgery, Faculty of Medicine, Medical University of Gdańsk, 80-210 Gdańsk, Poland; 3 Faculty of Medicine, Medical University of Gdańsk, 80-210 Gdańsk, Poland

**Keywords:** mitral valve surgery, machine learning, artificial intelligence, risk prediction, logistic regression, cardiac surgery, perioperative complications, explainable AI

## Abstract

**Background/Objectives:** Mitral valve surgery is associated with substantial perioperative heterogeneity and risk of postoperative complications. Although established risk scores such as EuroSCORE II provide population-level prognostic estimates, their performance may be limited in specific surgical populations and institutional settings. This pilot study aimed to develop and internally validate an interpretable center-specific machine learning model for perioperative risk stratification following mitral valve surgery and to explore its translational implementation through a prototype clinical application. **Methods:** A retrospective single-center study was conducted including 211 consecutive patients undergoing mitral valve surgery with ring implantation. Routinely available demographic, laboratory, and perioperative variables were evaluated as candidate predictors. The primary endpoint was a composite of major postoperative complications, including in-hospital mortality, stroke, conversion to sternotomy, and rethoracotomy. Predictive approaches included logistic regression, LASSO regression, and random forest classification. Internal validation was performed using 5-fold cross-validation and bootstrap resampling. Model explainability was assessed using regression coefficients and SHAP (SHapley Additive exPlanations) analysis. **Results:** The composite endpoint occurred in 34 patients (16.1%). In the complete-case final logistic regression model, apparent discrimination reached an AUC of 0.750 (95% CI 0.643–0.858), with a Brier score of 0.105. In the predefined train-test evaluation, the simplified logistic regression model achieved a test-set AUC of 0.67, while 5-fold cross-validation yielded a mean AUC of 0.75. LASSO regression achieved the highest cross-validated AUC (0.78), although with marked discrepancy between test-set and cross-validation performance, suggesting model instability. Across models, higher age, serum creatinine concentration, cardiopulmonary bypass duration, and cross-clamp time were associated with increased complication risk, whereas higher hemoglobin levels were associated with lower risk. **Conclusions:** This pilot study demonstrates the feasibility of developing interpretable center-specific machine learning models for perioperative risk stratification following mitral valve surgery. Simplified regression-based approaches provided clinically transparent predictions with moderate discriminatory performance, while penalized models showed potential for improved generalizability. Further multicenter validation is required before clinical implementation.

## 1. Background

Mitral valve surgery remains one of the most commonly performed procedures in contemporary cardiac surgery and is associated with substantial perioperative heterogeneity regarding patient characteristics, operative complexity, and postoperative outcomes. Despite ongoing advances in surgical techniques, perioperative care, minimally invasive approaches, and postoperative monitoring, major postoperative complications continue to represent an important clinical challenge.

Current perioperative risk assessment in cardiac surgery relies predominantly on generalized population-based scoring systems such as EuroSCORE II and the Society of Thoracic Surgeons (STS) risk models [1,2]. Although these tools provide valuable prognostic information at the population level, their predictive performance may be limited in specific procedural subgroups, including mitral valve surgery, particularly within evolving minimally invasive and institution-specific surgical pathways [3,4]. Furthermore, generalized risk calculators may insufficiently capture local perioperative practices, patient selection patterns, and center-specific procedural variability.

In recent years, machine learning approaches have gained increasing attention in perioperative medicine due to their potential ability to identify complex multidimensional relationships between clinical variables and postoperative outcomes [5,6]. Several studies have explored artificial intelligence-based prediction models in cardiac surgery; however, many available approaches remain limited by insufficient interpretability, lack of external validation, substantial model complexity, or reduced clinical applicability in routine perioperative workflows [5,7].

An additional challenge in perioperative artificial intelligence implementation involves balancing predictive performance with clinical interpretability. While highly complex machine learning algorithms may achieve strong predictive discrimination in selected datasets, limited transparency and “black-box” behavior remain substantial barriers to clinician trust and practical bedside implementation [5,8].

Institution-specific perioperative prediction models may represent a complementary approach to generalized population-based risk scores. Local surgical techniques, perioperative management strategies, postoperative care pathways, and institutional patient characteristics may substantially influence complication patterns and predictive relationships [3]. Consequently, continuously updated center-specific models may better reflect real-world perioperative practice within individual cardiac surgery centers.

The present pilot study aimed to develop and internally validate an interpretable institution-specific perioperative risk stratification model for patients undergoing mitral valve surgery using routinely available perioperative and laboratory variables. Additionally, the study explored practical translational implementation through the development of a lightweight browser-based prototype application designed to demonstrate the feasibility of rapid perioperative bedside risk estimation.

## 2. Methods

### 2.1. Study Design and Population

This retrospective single-center pilot study was conducted at the Department of Cardiac Surgery, University Clinical Centre in Gdańsk, Poland. The study included 211 consecutive adult patients undergoing mitral valve surgery with annuloplasty ring implantation. Both mitral valve repair and mitral valve replacement procedures were included. Clinical, perioperative, and postoperative data were retrospectively extracted from institutional databases and operative records. Evaluated variables included demographic characteristics (age, sex), laboratory parameters (hemoglobin concentration, serum creatinine), echocardiographic variables (left ventricular ejection fraction), operative variables (cardiopulmonary bypass time, aortic cross-clamp time, ring size, surgical access), functional status (NYHA class), and rhythm characteristics including atrial fibrillation status.

The primary aim of the study was to develop and internally validate an exploratory machine learning-based perioperative risk stratification model for major postoperative complications following mitral valve surgery. Due to the pilot and exploratory nature of the study, no formal sample size calculation was performed.

### 2.2. Variables and Preprocessing

Demographic, perioperative, echocardiographic, laboratory, and procedural variables routinely available in clinical practice were considered as candidate predictors. These included age, sex, hemoglobin concentration, serum creatinine concentration, cardiopulmonary bypass time, aortic cross-clamp time, left ventricular ejection fraction, ring size, atrial fibrillation status, NYHA functional class, surgical access, and procedural characteristics.

Continuous variables were inspected for implausible and non-physiological values. Cross-clamp time values equal to 0 min were considered invalid entries and treated as missing values. Similarly, creatinine values equal to 0 were excluded from analyses as implausible measurements.

Hemoglobin values were converted from string-based decimal formatting into numeric representation prior to analysis. Complete-case analysis was applied for model development. Missing data were limited for most variables, ranging from 0% to 1.9%, except for cross-clamp time (9.0%). Complete-case analysis retained 190 of 211 patients (90.0%) for final model development.

After exploratory model comparisons, a simplified clinically interpretable model including age, hemoglobin concentration, serum creatinine concentration, cardiopulmonary bypass time, and cross-clamp time was selected for the final analyses.

Because cardiopulmonary bypass time and cross-clamp time are intraoperative variables, the final model should be interpreted as a perioperative risk prediction model rather than a purely preoperative risk stratification tool.

### 2.3. Outcome Definition

The primary endpoint was the occurrence of major postoperative complications. A composite binary endpoint was created based on the presence of at least one severe postoperative adverse event recorded during hospitalization, including:

- in-hospital mortality;
- stroke;
- conversion to sternotomy;
- rethoracotomy.

The composite endpoint was coded as a binary variable (0 = no complication, 1 = at least one complication) and served as the dependent variable in all predictive analyses. In addition to the composite endpoint, the frequency of individual endpoint components and the occurrence of multiple events within individual patients were evaluated descriptively.

Composite cardiovascular endpoints are commonly used to improve statistical efficiency in studies with limited event counts, although heterogeneity between endpoint components should be considered when interpreting results [9].

### 2.4. Statistical Analysis

Descriptive statistics were calculated for all study variables. Continuous variables were summarized as mean ± standard deviation or median with interquartile range, depending on data distribution. Categorical variables were presented as counts and percentages.

All analyses were performed using Python 3.12.7 (Python Software Foundation, Wilmington, DE, USA) in JupyterLab 4.5.7. Data preprocessing was performed using pandas and NumPy, while predictive modeling was conducted using scikit-learn. Model explainability analyses were performed using SHAP.

### 2.5. Machine Learning Model Development

Several predictive approaches were evaluated, including logistic regression, penalized logistic regression (LASSO), and random forest classification. Because of the limited event count, model tuning was intentionally conservative, and the results of more complex models were interpreted descriptively rather than as clinically deployable prediction tools.

The dataset was divided into training and testing subsets using stratified random sampling (80/20 split). The final complete-case dataset contained 163 patients without complications (85.8%) and 27 patients with the composite endpoint (14.2%). Class imbalance was addressed using balanced class weighting.

Model discrimination was assessed using the area under the receiver operating characteristic curve (AUC-ROC). Additional model performance metrics included the Brier score, confusion matrix analysis, sensitivity, specificity, positive predictive value, negative predictive value, and calibration assessment using bootstrap validation. Calibration performance was evaluated using bootstrap-corrected calibration curves generated from 1000 bootstrap resamples.

To estimate model robustness and reduce the risk of overfitting, internal validation procedures included 5-fold cross-validation and bootstrap resampling.

Appropriate internal validation is considered essential for prediction model development, particularly in exploratory datasets with limited sample sizes [10].

### 2.6. Model Interpretability

Feature importance and explainability were evaluated using standardized logistic regression coefficients and SHAP (SHapley Additive exPlanations) analysis [11]. SHAP summary plots were generated to assess the relative contribution and directionality of variables influencing model predictions. Emphasis on explainability and model transparency is increasingly recognized as an important prerequisite for clinical implementation of artificial intelligence systems in medicine [12].

A lightweight browser-based prototype application was developed solely as a proof-of-concept demonstration of potential translational implementation. The prototype is not intended for clinical use and requires independent validation before any practical deployment can be considered.

### 2.7. Ethical Considerations

The study was conducted in accordance with institutional standards for retrospective observational research using anonymized routinely collected clinical data.

## 3. Results

### 3.1. Study Population

**Figure 1:**
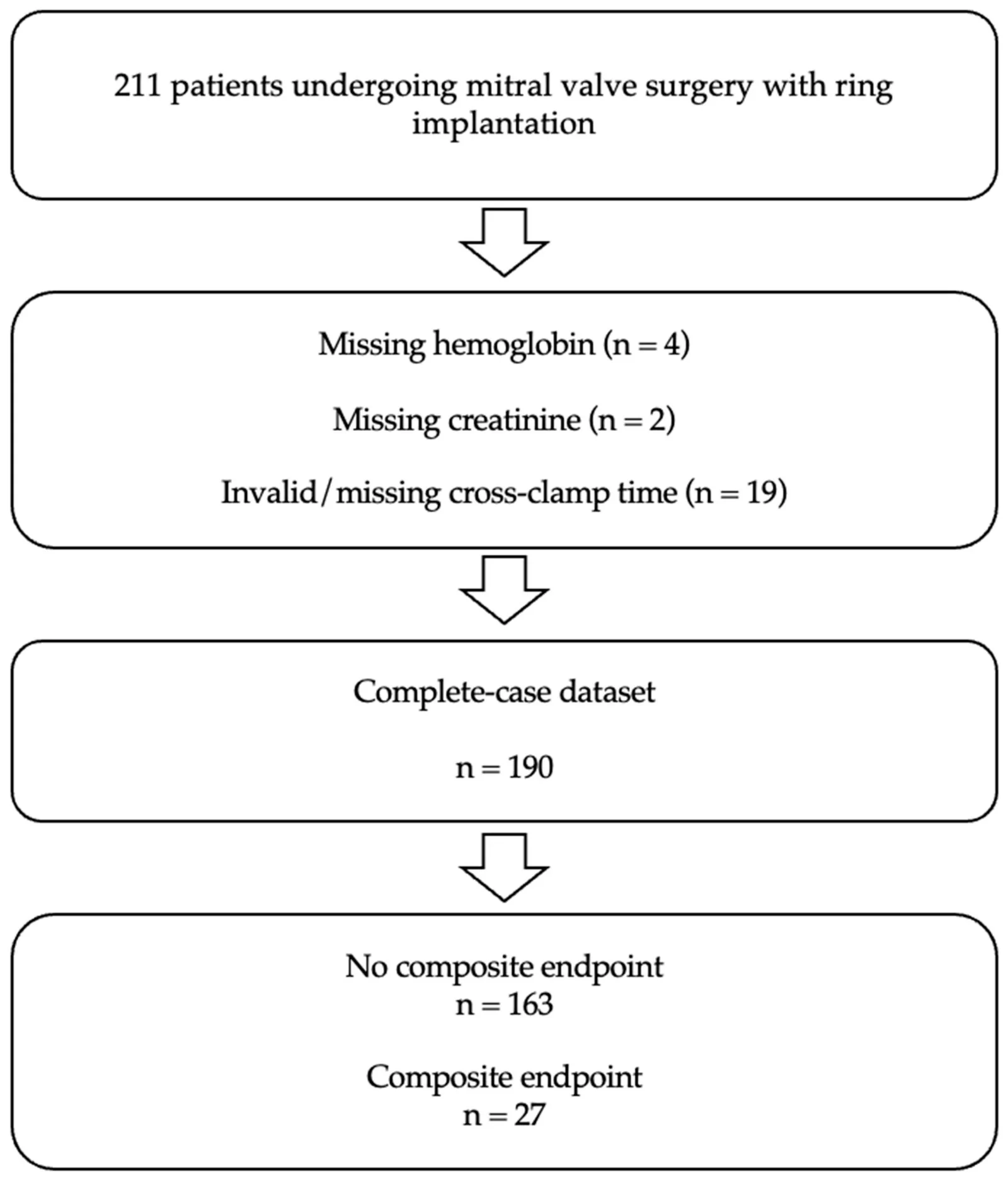
Patient selection flowchart showing inclusion criteria, exclusions due to missing data, and derivation of the final complete-case analytic dataset used for model development.

The final study cohort consisted of 211 patients undergoing mitral valve surgery with ring implantation. Baseline demographic, perioperative, and postoperative characteristics are summarized in Table 1. Patient selection and derivation of the final analytic dataset are presented in Figure 1.

The mean age of the cohort was 60.4 ± 13.7 years, and 57.8% of patients were male. Mean left ventricular ejection fraction was 53.8 ± 8.9%. Mean cardiopulmonary bypass time was 149.8 ± 47.5 min, while median cross-clamp time was 92 min (IQR 72–109).

Mitral valve repair was performed in 72.5% of procedures, whereas mitral valve replacement accounted for 27.5% of cases. Endoscopic access was used in 17.1% of operations.

Postoperative atrial fibrillation occurred in 55.9% of patients. The observed in-hospital mortality rate was 4.7%, while stroke occurred in 1.9% of patients. Additional postoperative outcomes included rethoracotomy in 8.1% of cases, conversion to sternotomy in 2.8%, and permanent pacemaker implantation in 3.3% of patients.

The composite endpoint of major postoperative complications, defined as the occurrence of in-hospital mortality, stroke, conversion to sternotomy, or rethoracotomy, occurred in 34 patients (16.1%). Among patients experiencing the composite endpoint, 31 experienced a single adverse event, whereas 3 patients experienced more than one endpoint component during hospitalization.

### 3.2. Predictive Model Performance

**Figure 2:**
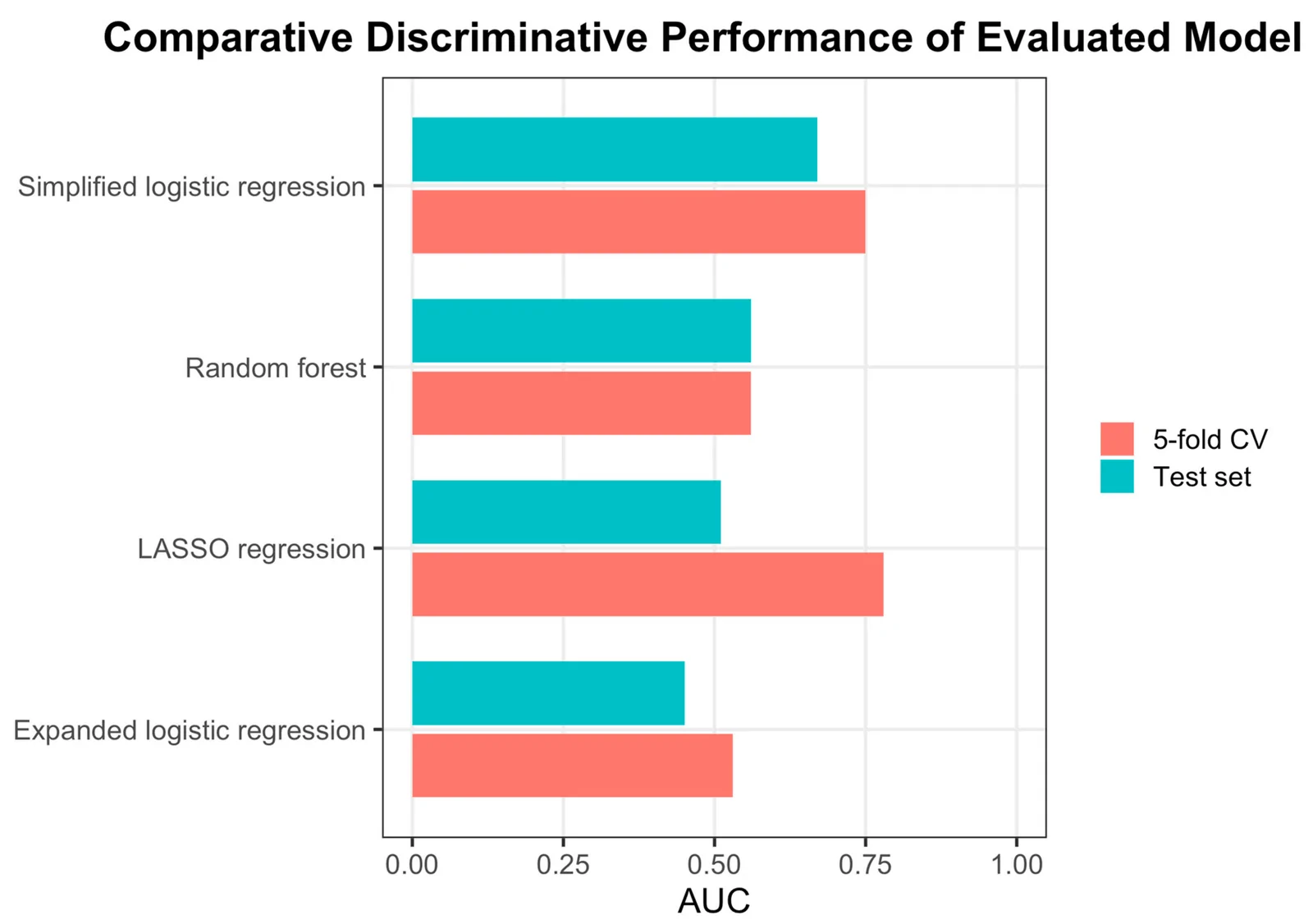
Comparative discriminatory performance of the evaluated prediction models. Test-set and 5-fold cross-validation AUC values are presented for the simplified logistic regression, expanded logistic regression, random forest, and LASSO regression models.

**Figure 3:**
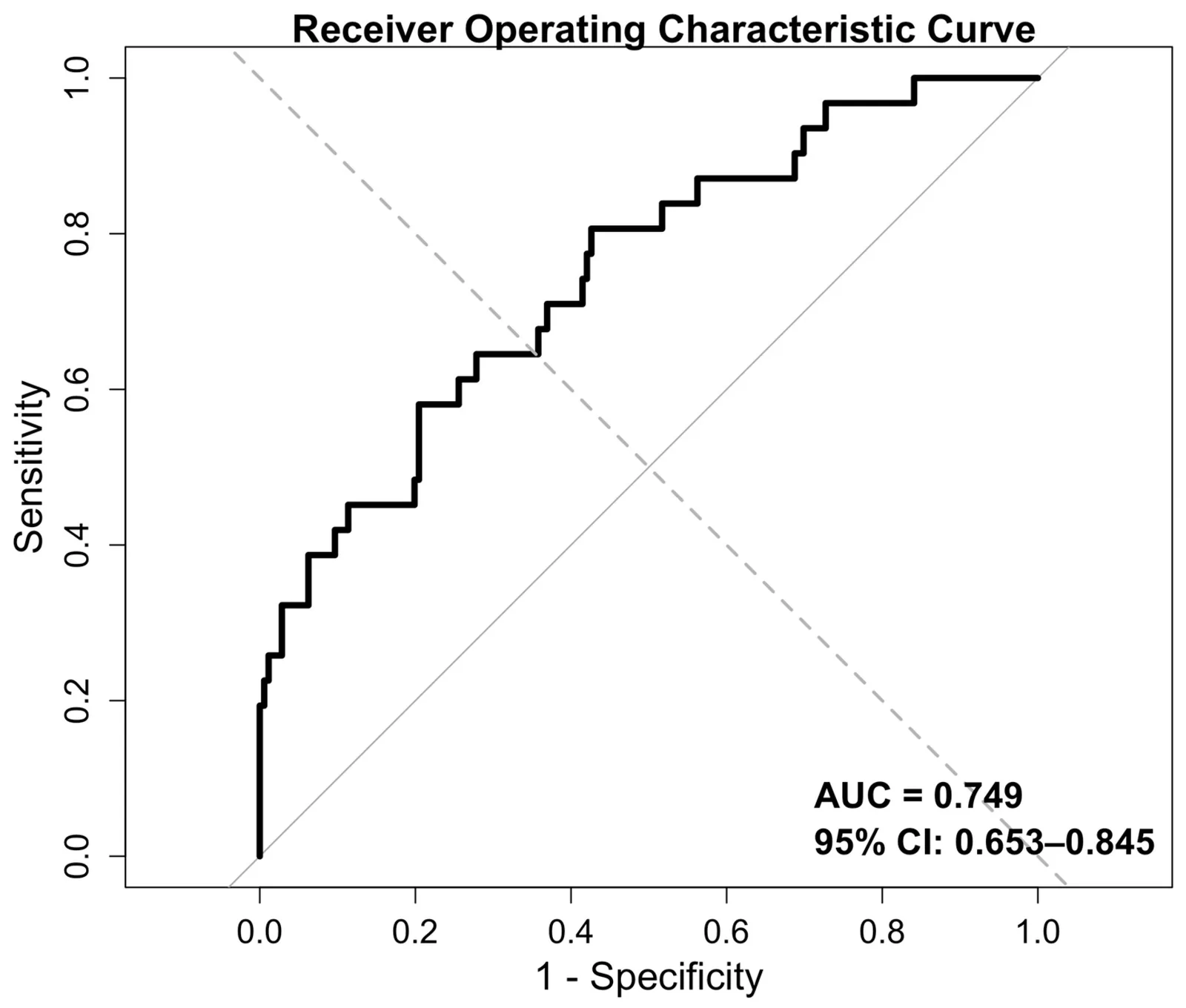
Receiver operating characteristic (ROC) curve of the final logistic regression model predicting major postoperative complications after mitral valve surgery. The model achieved an AUC of 0.75 (95% CI 0.65–0.85).

Comparative performance of evaluated predictive models is presented in Table 2 and Figure 2. Among the evaluated approaches, the simplified logistic regression model demonstrated the most favorable balance between interpretability and predictive performance. In the predefined train-test evaluation, the model achieved a test-set AUC of 0.67, while 5-fold cross-validation yielded a mean AUC of 0.75 (Table 2 and Figure 2). In the final complete-case model fitted to the analytic dataset (*n* = 190), apparent discrimination reached an AUC of 0.750 (95% CI 0.643–0.858), with a Brier score of 0.105 (Figure 3). These differences between evaluation approaches indicate moderate model instability and should be interpreted in the context of the limited number of outcome events. Bootstrap calibration demonstrated acceptable overall calibration, although model performance remained limited by the relatively small number of outcome events.

Expanded multivariable models containing larger numbers of perioperative and categorical predictors demonstrated lower generalizability and reduced predictive performance, suggesting overfitting in the context of a relatively small single-center cohort.

Random forest classification models demonstrated lower overall discrimination and greater instability across validation folds compared with regularized regression approaches.

Interestingly, LASSO regression achieved a mean cross-validated AUC of 0.78 (SD 0.18) despite lower single test-set discrimination. The penalized model retained only a limited subset of predictors, with cardiopulmonary bypass time emerging as the dominant retained variable, suggesting that sparse regularized models may improve generalizability in smaller perioperative datasets.

### 3.3. Feature Importance and Explainability

Across evaluated models, higher age, serum creatinine concentration, cardiopulmonary bypass time, and cross-clamp time were consistently associated with increased predicted complication risk, whereas higher hemoglobin concentration was associated with lower predicted risk.

Multivariable logistic regression identified cardiopulmonary bypass time as the only statistically significant independent predictor of major postoperative complications. Increasing age demonstrated a trend toward significance (Table 3). Hemoglobin concentration, serum creatinine concentration, and cross-clamp time were not independently associated with the composite endpoint.

**Figure 4:**
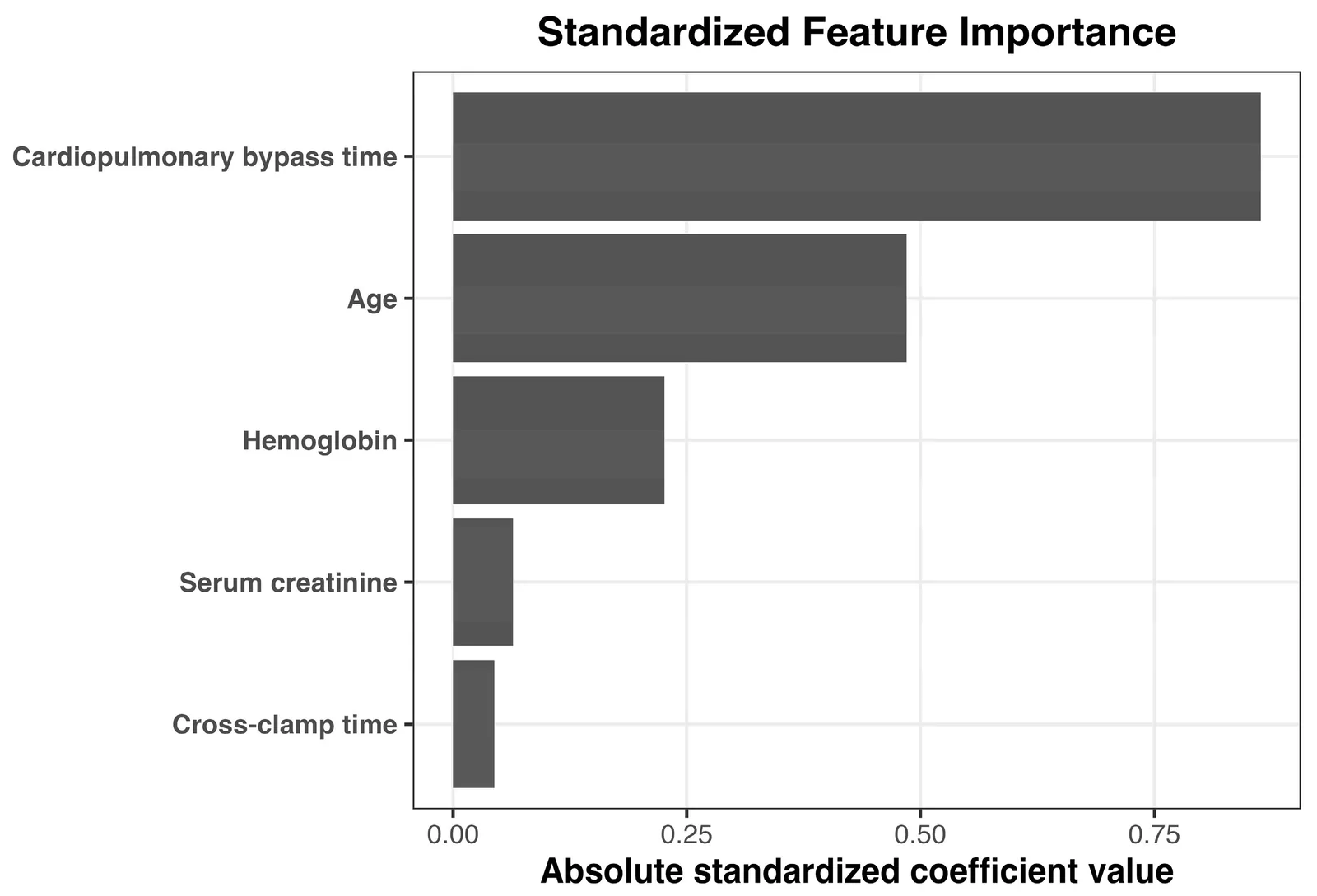
Standardized feature importance in the final logistic regression model expressed as absolute standardized regression coefficient values. Cardiopulmonary bypass time demonstrated the strongest contribution to model predictions, followed by age and hemoglobin concentration. Serum creatinine and cross-clamp time showed comparatively smaller contributions.

**Figure 5:**
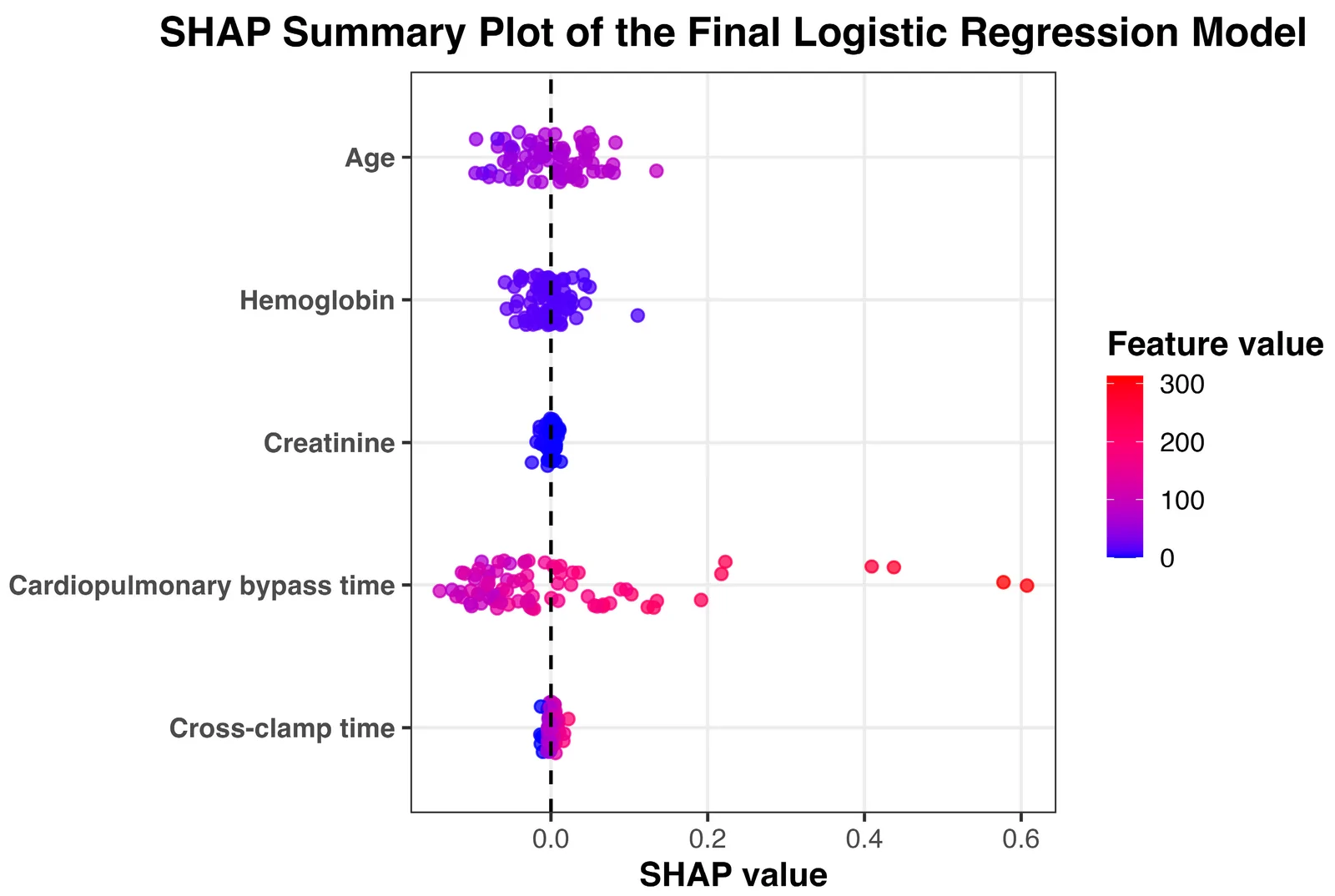
SHAP summary (beeswarm) plot showing the contribution of predictor variables to the final logistic regression model. Each point represents an individual patient. Positive SHAP values indicate an increased predicted risk of major postoperative complications, whereas negative values indicate lower predicted risk. Point color indicates the original feature value.

Regression-based feature importance is presented in Figure 4, while SHAP-based explainability analysis is shown in Figure 5. Standardized coefficient-based feature importance and SHAP-based explainability provided complementary perspectives on model interpretation. Both approaches identified cardiopulmonary bypass time as the most influential predictor, followed by age and hemoglobin concentration, while serum creatinine and cross-clamp time showed smaller contributions.

### 3.4. Calibration and Internal Validation

**Figure 6:**
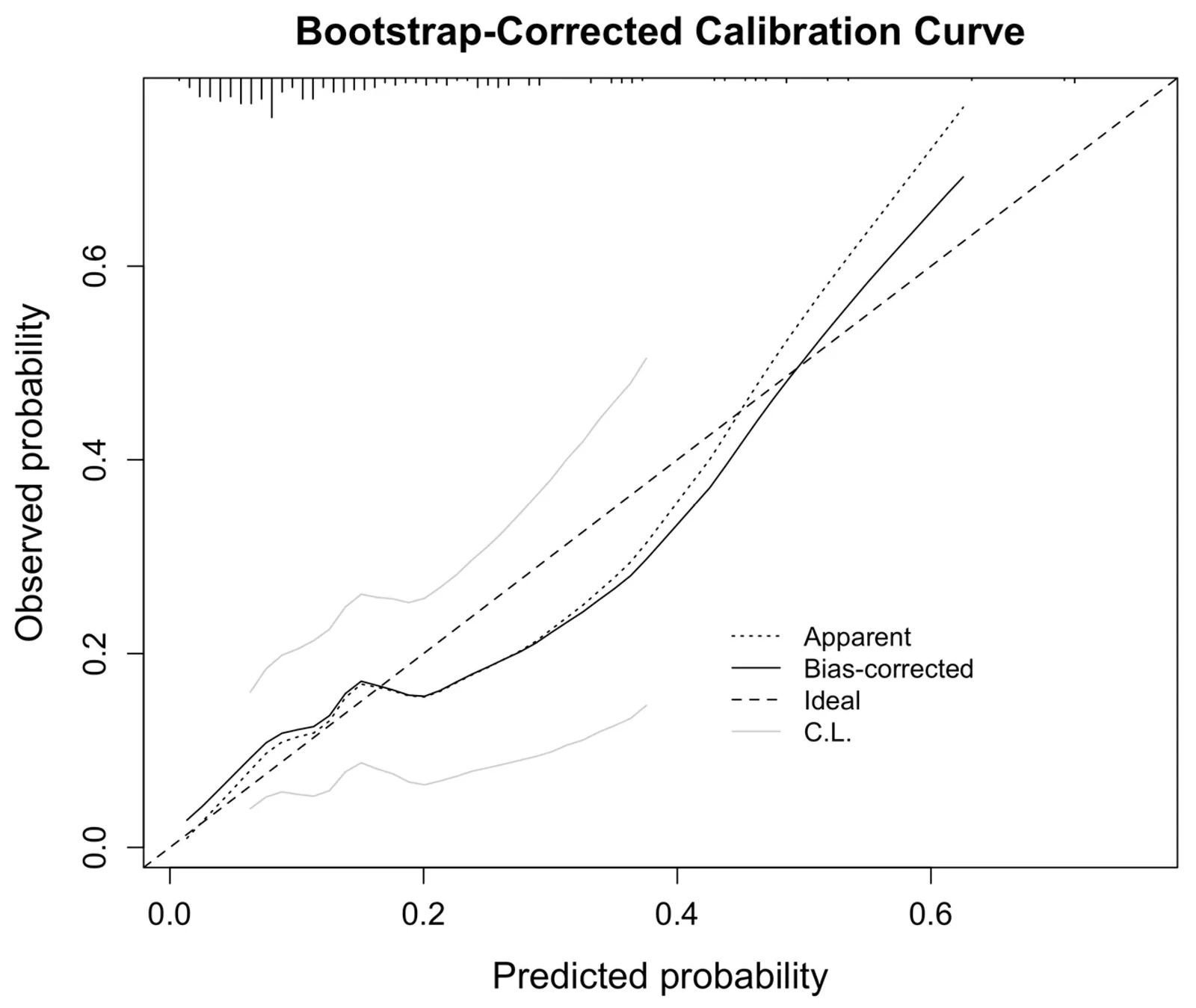
Bootstrap-corrected calibration curve of the final logistic regression model predicting major postoperative complications after mitral valve surgery. Bootstrap calibration was performed using 1000 resamples.

Calibration analysis demonstrated satisfactory agreement between predicted and observed event probabilities (Figure 6). Bootstrap calibration yielded a mean absolute calibration error of 0.031 and a mean squared error of 0.00138. Model calibration remained less stable at higher predicted probability ranges, likely reflecting the limited number of adverse events [13].

Apparent calibration was excellent, with a calibration intercept of 0.00 and a calibration slope of 1.00. Because calibration was assessed on the development dataset, these estimates should be interpreted as measures of apparent rather than externally validated calibration.

Bootstrap validation demonstrated moderate overall robustness but relatively broad confidence intervals, reflecting the exploratory nature of the study and limited event count.

Threshold-dependent analysis demonstrated that a probability cut-off of 0.15 yielded the highest balanced accuracy (0.692), corresponding to a sensitivity of 63.0%, specificity of 75.5%, positive predictive value of 29.8%, and negative predictive value of 92.5%. Lowering the classification threshold improved sensitivity for detecting complications at the cost of increased false-positive classifications consistent with known trade-offs in ROC-based threshold optimization [14].

### 3.5. Prototype Application

A lightweight browser-based prototype application (“Mitral Valve Risk AI”) was developed solely as a proof-of-concept research tool to illustrate potential technical implementation of the model. The prototype should not be interpreted as a clinically validated decision-support system.

## 4. Discussion

The present pilot study demonstrates the feasibility of developing institution-specific perioperative risk stratification models for patients undergoing mitral valve surgery using routinely available clinical and laboratory variables. Despite the relatively limited cohort size, several machine learning and statistical approaches demonstrated moderate predictive capability for major postoperative complications.

The discriminatory performance observed in the final model (AUC 0.75) should be interpreted as moderate but clinically meaningful, particularly given the relatively small sample size and limited number of adverse events. The observed performance is comparable to several previously reported perioperative prediction models developed using routinely available clinical variables.

Interestingly, model performance varied substantially depending on the degree of model complexity and regularization. While the simplified logistic regression model demonstrated the best balance between interpretability, clinical transparency, and overall consistency, LASSO regularization achieved the highest cross-validated performance. Notably, the present findings suggest that increased algorithmic complexity did not translate into superior performance, reinforcing previous evidence that conventional regression-based approaches may remain competitive with more complex machine learning methods in smaller clinical datasets. This finding suggests that feature reduction and penalization may improve model parsimony, although prediction models developed in smaller datasets may remain vulnerable to instability and overfitting [15,16].

In contrast, expanded multivariable models and random forest approaches demonstrated lower stability and weaker overall discrimination. These findings are consistent with evidence suggesting that higher-complexity machine learning algorithms do not universally outperform simpler statistical approaches, particularly in relatively small clinical datasets vulnerable to overfitting [15,16]. This observation is consistent with the present study, in which the best-performing and most clinically interpretable model was a conventional logistic regression model.

Several predictors demonstrated clinically plausible associations with postoperative risk. Older age, impaired renal function, prolonged cardiopulmonary bypass exposure, and longer cross-clamp time are established markers of increased perioperative burden and physiological vulnerability in cardiac surgery populations [17,18]. In contrast, higher hemoglobin concentration appeared protective, potentially reflecting greater baseline physiological reserve and reduced perioperative frailty; this is consistent with evidence linking preoperative anemia with worse outcomes after cardiac surgery [19].

An important aspect of the present study is the emphasis on explainability and clinical interpretability. Rather than relying exclusively on black-box algorithms, the study incorporated transparent coefficient-based modeling alongside SHAP explainability analyses [11]. This approach may be particularly relevant in perioperative medicine, where clinician trust, transparency, reporting quality, and interpretability remain major barriers to broader implementation of artificial intelligence-based tools [5,8,20,21].

The study additionally explored practical translational implementation through the development of a lightweight browser-based prototype application. Although preliminary, this proof-of-concept demonstrates the technical feasibility of developing institution-specific perioperative prediction tools; however, clinical integration would require prospective validation, external evaluation, and comparison with established risk scores. Similar recent work in cardiac surgery has highlighted the translational potential of machine-learning-based prediction models and clinically oriented digital applications [21,22].

The findings may support the broader concept of center-specific perioperative risk stratification systems in cardiac surgery. Institutional differences in surgical techniques, perioperative management, patient selection, and postoperative care may substantially influence complication patterns and predictive relationships. Consequently, locally developed and continuously updated predictive models may provide clinically relevant complementary information beyond generalized population-based risk scores [3,4,21].

## 5. Limitations

Several limitations should be acknowledged. First, this was a retrospective single-center study conducted at a tertiary academic cardiac surgery center, which may limit the generalizability of the findings and introduce institution-specific bias related to patient selection, surgical strategy, and perioperative management.

Additionally, the number of events per predictor remained relatively limited, which may have affected coefficient stability and confidence interval precision despite the use of bootstrap validation procedures.

Second, the cohort size and number of adverse events were relatively limited, increasing the risk of model instability and overfitting. The final complete-case analytic dataset contained 190 patients and 27 outcome events, which limits statistical power and increases susceptibility to model instability despite internal validation procedures. Smaller clinical datasets may reduce the reliability and transportability of predictive models, particularly when multiple candidate predictors are evaluated [23]. Although internal validation procedures including cross-validation and bootstrap resampling were performed, the models should still be interpreted as exploratory and hypothesis-generating.

Detailed perioperative anticoagulation and antiplatelet management data were not consistently available and therefore could not be incorporated into model development. Furthermore, ring size was analyzed as an absolute surgical measurement and was not normalized to body surface area.

Importantly, cardiopulmonary bypass time and cross-clamp time become available only intraoperatively. Therefore, the present model should be interpreted as a perioperative or intraoperative risk prediction model rather than a purely preoperative risk stratification tool.

Third, some variables required manual preprocessing and cleaning, including removal of implausible entries. Additionally, the composite endpoint was based on retrospectively collected institutional data and may not fully capture the heterogeneity of clinically relevant postoperative complications.

Moreover, the composite endpoint combined outcomes with different clinical mechanisms and severity, including mortality, stroke, conversion to sternotomy, and rethoracotomy; therefore, the model should be interpreted as predicting a broad adverse perioperative course rather than a single mechanistically homogeneous complication.

Direct comparison with EuroSCORE II or STS risk scores was not possible because these scores were not consistently available in structured form for all patients in the retrospective dataset. Similarly, the retrospective structure of the dataset limited complete reconstruction of all variables required for established risk scores or endpoint-specific prediction models. Future studies should prospectively collect established risk scores to enable direct comparison, calibration assessment, and evaluation of incremental predictive value.

Finally, external multicenter validation will be necessary before any clinical implementation can be considered.

## 6. Conclusions

This pilot study demonstrates the technical feasibility of developing interpretable institution-specific perioperative risk prediction models for mitral valve surgery using routinely available clinical variables. Simplified logistic regression models provided strong interpretability and moderate discriminatory performance, although model stability remained limited by the small number of outcome events. Further multicenter validation and prospective refinement are required before clinical deployment.

## Figures and Tables

**Table 1:** Baseline characteristics of the study cohort.

Variable	Value
Age, years	60.4 ± 13.7
Hemoglobin, g/dL	13.7 ± 1.6
Creatinine, mg/dL	1.0 ± 0.4
Cardiopulmonary bypass time, min	149.8 ± 47.5
Cross-clamp time, min	93.7 ± 28.8
Ejection fraction, %	53.8 ± 8.9
Ring size	33.5 ± 4.2
Male sex	122 (57.8%)
Endoscopic access	36 (17.1%)
Mitral valve repair	153 (72.5%)
Mitral valve replacement	58 (27.5%)
Preoperative atrial fibrillation	98 (46.4%)
Postoperative atrial fibrillation	118 (55.9%)
Rethoracotomy	17 (8.1%)
Stroke	4 (1.9%)
Conversion to sternotomy	6 (2.8%)
Pacemaker implantation	7 (3.3%)
In-hospital mortality	10 (4.7%)
Major postoperative complication composite endpoint	34 (16.1%)

**Table 2:** Comparative performance of predictive models.

Model	Test AUC	Mean CV AUC	Comment
Logistic regression (simplified)	0.67	0.75	Interpretable model with stable performance
Logistic regression (expanded)	0.45	0.53	Lower generalizability and possible overfitting
Random forest	0.56	0.56	Moderate and less stable discrimination
LASSO regression	0.51	0.78	Strong cross-validation performance with sparse feature selection

**Table 3:** Final logistic regression model predicting major postoperative complications.

Predictor	OR	95% CI	*p*-Value
Age	1.04	1.00–1.08	0.069
Hemoglobin	0.87	0.66–1.15	0.324
Creatinine	0.85	0.29–2.17	0.727
Cardiopulmonary bypass time	1.02	1.01–1.03	0.001
Cross-clamp time	1.00	0.99–1.01	0.847

## Data Availability

The data presented in this study are available upon request from the corresponding author due to privacy and institutional restrictions related to patient-level clinical data.
